# Robot-assisted Partial Nephrectomy with and without Mixed Reality - REALITATEM study

**DOI:** 10.1590/S1677-5538.IBJU.2025.0463

**Published:** 2025-10-30

**Authors:** Dorival Manrique Duarte, Pietro Waltrick Brum, Milton Berger, Andrey Kowalski, Brasil Silva, André Kives Berger

**Affiliations:** 1 Universidade Federal do Rio Grande do Sul Porto Alegre RS Brasil Universidade Federal do Rio Grande do Sul – UFRS, Porto Alegre, RS, Brasil; 2 Hospital Moinhos de Vento Porto Alegre RS Brasil Hospital Moinhos de Vento, Porto Alegre, RS, Brasil; 3 Complexo Hospitalar Santa Casa Porto Alegre RS Brasil Complexo Hospitalar Santa Casa - Porto Alegre, RS, Brasil; 4 Universidade Vale do Taquari Lajeado RS Brasil Universidade Vale do Taquari - Lajeado, RS, Brasil

**Keywords:** Nephrectomy, Robotic Surgical Procedures, Augmented Reality

## Abstract

**Purpose:**

This randomized clinical trial (RCT) was developed to analyze the efficacy of using Mixed Reality (MIXREAL), the combination of virtual (VR) and augmented realities (AR), in robot-assisted partial nephrectomy (RAPN).

**Materials and Methods:**

Forty-five patients with renal masses (RM) were allocated to RAPN with or without use of MIXREAL, Realitatem Group (RG) and Control Group (CG), respectively.

**Results:**

Analyses indicated statistically significant difference in ischemia time favoring RG (p = 0.045), with a mean difference of 3.8 minutes. Classically, the limit widely accepted as suitable for ischemia time is 20-25 minutes, but every 1 minute saved may reduce renal injury. Analyses also indicated statistically significant difference in decision for selective clamping favoring RG (p = 0.013); main renal artery clamping globally exposes the renal parenchyma to ischemia. The percentage of residual parenchyma after surgery is also an important variable to renal function recovery, and this study presented a trend towards the enucleation technique being facilitated in the RG. No difference was detected regarding complication rate. Despite those results, no difference was detected in both short and long-term renal function outcomes. The small sample is an important drawback.

**Conclusion:**

This RCT demonstrates the feasibility and safety of MIXREAL in RAPN, as well as its potential to support intraoperative decision-making. It represents the first RCT evaluating MIXREAL in RAPN. Larger studies with longer follow-up are needed to confirm potential functional benefits.

## INTRODUCTION

Kidney cancer has had a rising incidence (1) and PN is the standard of care for RM stage cT1/2 (2); PN may be supported by a variety of tools, such as three-dimensional (3D) models (3). VR is defined as an artificial 3D visual environment and AR, as virtual objects superimposed on the real world (4); MIXREAL is the association between VR and AR (5).

The first clinical experience using AR in a PN was in 2008 (6), and since then, clinical trials of 3D assisted minimally invasive PN have been developed, such as the first trial evaluating both AR and VR in videolaparoscopic PN (7), and the first RCT evaluating VR in RAPN (8); subsequently, Porpiglia et al. and Li et al. published trials of RAPN using exclusively AR (9, 10).

We aim to assess perioperative outcomes of RAPN with the use of MIXREAL. To our knowledge, this is not only the first study in Latin America to employ MIXREAL in minimally invasive PN, but also the first RCT worldwide to combine VR and AR in the context of RAPN. We hypothesize that as well as the pioneering studies mentioned, we will demonstrate primarily feasibility and safety of MIXREAL in RAPN and can expect improvements in perioperative outcomes.

## MATERIALS AND METHODS

After approval by the ethics commission (IRB: 66791623.8.0000.5330) of Moinhos de Vento Hospital (Porto Alegre, Rio Grande do Sul), patients from hospital's clinic with solid or cystic RM requiring PN were prospectively randomized (protocol NCT06903260) to either RG or CG in a 1:1 ratio; the random sequence was generated using a computer-based random number generator. Patients were blinded to the group allocation. Exclusion criteria comprised patients with metastatic disease, RM staged ≥ cT3 or cN1, tumors with an infiltrative growth pattern, or lesions suspected of urothelial histology.

**Figure 1:**
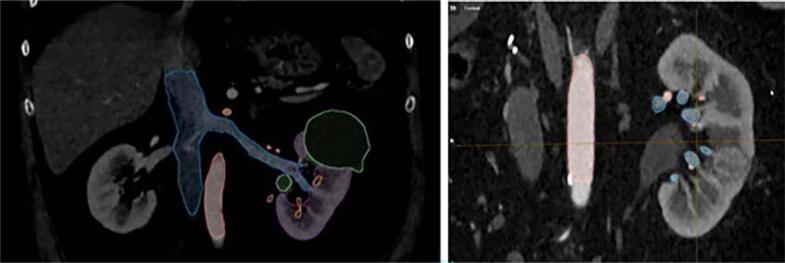
Images exemplifies the marking of anatomical structures on the image of angio-TC through Brainlab® software; note that each category has a different color and that the marking has to be done manually in many cuts of a single window (coronal, in this instance).

**Figure 2:**
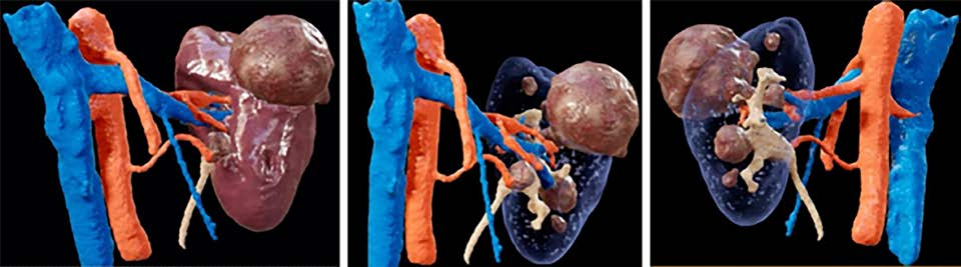
Examples of virtual reality through the coronal axis, depicting venous, arterial and collecting systems, parenchyma and the tumors: 2a keeps the parenchyma, evidencing in an anterior view only the exophytic portions of the tumors; in 2b the parenchyma was removed, evidencing the endophytic portions and its relation to vessels and collecting system; 2c has the same purpose of 2b, but through a posterior view.

**Figure 3:**
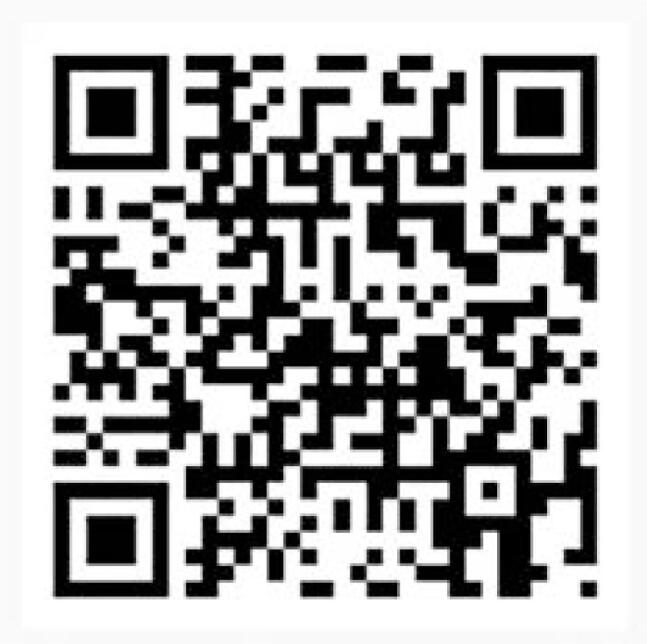
QR code to access the video of the complete 3D reconstruction of the case presented in figure 2, from the anatomical marking to the final virtual reality result.

A computerized tomography (CT) angiography was performed within one month from the surgery. The images were exported in DICOM (Digital Imaging and Communications in Medicine) and applied in Brainlab Elements® software (Brainlab AG, Munich, Germany), where the images and 3D drawing were rendered to obtain the VR (Figures 1, 2 and 3). Planned cases were available via cloud services for immediate use in the operating room (www.brainlab.com).

**Figure 4:**
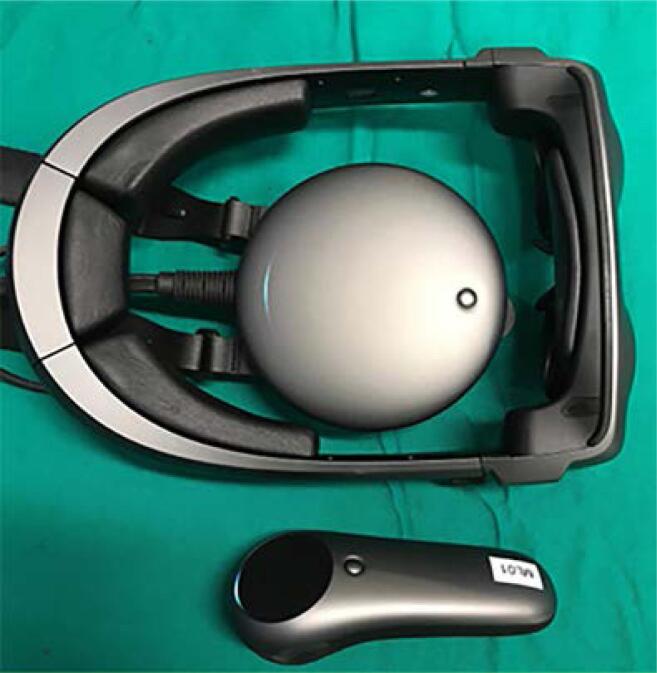
The Magic Leap 1 is an Augmented Reality device made up of three main components. The Lightwear is the headset that projects 3D digital images into the real world, along with sensors and eye-tracking for environment and user interaction. The Lightpack is a small, wearable processing unit that handles all computing tasks, battery, and runs the device's operating system. The Control is a handheld controller with a touchpad, buttons, motion sensors, and haptic feedback, allowing precise interaction with virtual elements.

**Figure 5:**
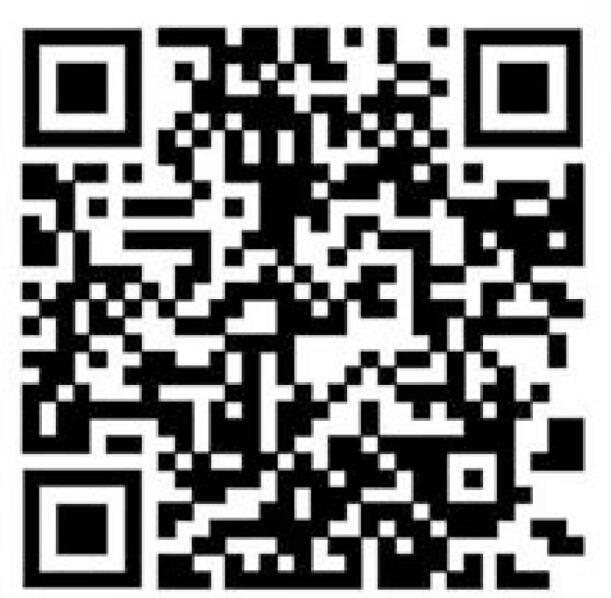
QR code to access a video of the theatre room during the intraoperative of a case from our study, through the lenses of the Magic Leap Goggle, depicting how virtual objects can be superimposed on the real world.

All surgeries were robot-assisted, transperitoneal, and executed at Moinhos de Vento Hospital, from August 2022 to January 2024 by 8 urologists with experience in RAPN. AR was obtained through the Magic Leap 1 goggle (Magic Leap Inc., Plantation, FL, USA) (Figures 4 and 5).

Besides tumor and patient's baseline characteristics and intra-operative data, such as vessel clamping, ischemia time, estimated blood loss (EBL), use of hemostatic agents and excision technique, post-operative data, such as renal function, pathology results, complication rate and hospital staying, were also recorded. Our primary outcome was ischemia time.

Data were analyzed using the Statistical Package for the Social Sciences (SPSS), version 27.0 (IBM Corp., Armonk, NY, USA). Descriptive statistics were presented as mean and standard deviation (SD) or median and interquartile range (IQR) for continuous variables, and as frequencies for categorical variables; comparisons were made between GR and GC through non-parametric tests Mann-Whitney, Chi-square (CH2) and, when necessary, Fisher's exact test. A GEE (Generalized Estimating Equations) model was fitted to evaluate the effect of treatment over time on the delta of serum creatinine and glomerular filtration rate (GFR). For all analyzes performed, it was adopted the 95% confidence interval and the significance level of 5% (p ≤ 0.05).

The sample was calculated using the Risk Calc software. Assuming a difference in mean ischemia rate between treatment groups to be 3.9 minutes (11), an expected population SD to be 3.23 (10) and a clinically relevant difference to be of 1 minute (12), to achieve 80% power (i.e., 1−β=0.8) at the level of significance of 5% (α=0.05) with equal allocation (i.e., k=1) and dropout rate of 5%, a total sample of at least 34 patients, divided into two groups, would be required.

## RESULTS

Regarding sociodemographic data (Table-1), the groups were homogeneous, with a predominance of males, in their 60s, overweight and moderately comorbid, according to Charlson comorbidity index (CCI). Regarding tumor data (Table-1), most were solid, with mean size range of 3.1-3.4 cm, and of intermediary complexity, according to RENAL Nephrometry Score.

**Table 1:** Sociodemographic and preoperative data, distributed across groups.

Variable		RG (n=20)	CG (n=25)	p
Gender, n (%)	Female	7 (35.0)	7 (28.0)	
	Male	13 (65.0)	18 (72.0)	
Age, *Mean (SD)*		60.2 (12.0)	60.4 (15.4)	
Ethnicity, n (%)	White	17 (85.0)	25 (100.0)	0.045
	Black	3 (15.0)	-	
BMI (kg/m^2^), *Mean (SD)*		28.6 (4.1)	26.8 (5.1)	
Family history of kidney cancer, n (%)	Yes	2 (10.0)	1 (4.0)	
				
Previous abdominal surgery, n (%)	Yes	8 (40.0)	7 (28.0)	
	Nephrectomy	4	-
Renal Lesion type, n (%)	Nodule	17 (85.0)	21 (84.0)	
	Cyst	3 (15.0)	4 (16.0)
	Bosniak III	2	3
	Bosniak IV	1	1
Renal Lesion size (cm), *Mean (SD)*		3.1 (1.0)	3.4 (2.4)	
Renal Lesion Laterality, n (%)	Left	10 (50.0)	14 (56.0)	
	Right	8 (40.0)	10 (40.0)
	Both	2 (10.0)	1 (4.0)
Multiple lesions	Yes	6 (30.0)	2 (8.0)	
	Two	4	2
	Three or more	2	-
CCI, *Mean (SD)*		4.0 (1.9)	4.4 (2.7)	
R.E.N.A.L. Score, n (%)	Low (≤6)	5 (25.0)	11 (44.0)	
	Intermediary (7-9)	9 (45.0)	12 (48.0)
	High (≥10)	6 (30.0)	2 (8.0)
ASA Score, n (%)	I	1 (5.0)	-	
	II	17 (85.0)	20 (80.0)
	III	2 (10.0)	5 (20.0)

RG = Realitatem group; CG = Control group; n = number of patients; *p* = statistical significance; Me = mean; SD = standard deviation; BMI = body mass index; CCI = Charlson comorbidity index; ASA = American Society of Anesthesiologists.

Perioperative data are shown in Table-2. Regarding ischemia, mean ischemia time was 14.6 and 18.4 minutes in RG and CG, respectively (p = 0.045), and in RG there were 5 selective clamping cases, while in the GC, none (p = 0.013). Off clamp procedure occurred in 40% and 28% of RG and CG surgeries, respectively (p = 0.527).

**Table 2:** Perioperative data, distributed across groups.

Variable		RG (n=20)	CG (n=25)	p
TST (min), *Mean (SD)*		181.0 (59.0)	153.0 (68.1)	
Off-Clamp, n (%)	Yes	8 (40.0)	7 (28.0)	
Ischemia Time (min), *Mean (SD)*		14.6 (12.6)	18.4 (8.9)	0.045
Selective Clamping, n (%)	Yes	5 (25.0)	-	0.013
EBL (mL), *Mean (SD)*		264.6 (223.6)	138.0 (147.5)	
Use of hemostatic agents, n (%)	Yes	19 (95.0)	22 (88.0)	
Red blood cell transfusion, n (%)	Yes	0 (0)	1 (4.0)	
Excision technique, n (%)	Wedge resection	5 (25.0)	5 (20.0)	
	Enucleoresection	7 (35.0)	15 (60.0)	
	Enucleation	8 (40.0)	5 (20.0)	
Conversion to RN, n (%)	Yes	0 (0)	2 (8.0)	
	Yes	3 (15.0)	1 (4.0)	
Perioperative complication, n (%)	I	2	-	
*Clavien-Dindo Classification	II	1	1	
HS (days), *Mean (SD)*		3.3 (2.5)	2.7 (1.6)	
Staging, n (%)	pT1a	13 (65.0)	16 (64.0)	
	pT1b	2 (10.0)	3 (12.0)	
	pT2	-	2 (8.0)	
	pT3	2 (10.0)	-	
	Benign	3 (15.0)	4 (16.0)	
				
Malignant variants, n (%)	Clear cell	10 (50.0)	18 (72.0)	
	Papillary	6 (30.0)	1 (4.0)	
	Chromophobe	1 (5.0)	2 (8.0)	
Baseline Cr, *Mean (SD)*		1.17 (0.47)	0.99 (0.30)	
30 days PO ΔCr, *Mean (SD)*		0.123 (0.51)	0.37 (0.36)	
*missing (n)		2	4	
90 days PO ΔCr, *Mean (SD)*		-0.04 (0.71)	0.2 (0.44)	
*missing (n)		7	15	
180 days PO ΔCr, *Mean (SD)*		-0.17 (0.76)	-0.02 (0.34)	
*missing (n)		8	17	
Baseline GFR, *Mean (SD)*		74.51 (34.09)	83.32 (27.76)	
30 days PO ΔGFR, *Mean (SD)*		-8.58 (24.9)	-17.64 (17.29)	
*missing (n)		2	4	
90 days PO ΔGFR, *Mean (SD)*		-2.18 (25.39)	-6.71 (20.1)	
*missing (n)		7	15	
180 days PO ΔGFR, *Mean (SD)*		-5.28 (22.97)	1.44 (15.54)	
*missing (n)		8	17	

RG = Realitatem group; CG = Control group; n = number of patients; p = statistical significance; Me = mean; SD = standard deviation; EBL = estimated blood loss; RN = radical nephrectomy; HS = hospital staying; Cr = creatinine in mg/dL; PO = postoperative; GFR = glomerular filtration rate in mL/min/1,73m2; Δ = difference from baseline.

The EBL was 264.6 mL and 138.0 mL in RG and CG, respectively (p = 0.085). Regarding hemostatic agent, 95% of RG and 88% of CG used it (p = 0.394), and only 1 patient underwent transfusion of red blood cells, from CG (p = 0.556). As for the resection technique, enucleation occurred more frequently in the RG (40 vs 20%; p = 0.288). Conversion to radical nephrectomy (RN) occurred only in CG, in 2 cases, where the tumor was hilar and the main renal vein drained directly from the tumor. No case was converted to open surgery.

Regarding complication, there was no difference between groups, and most were Clavien-Dindo grade I. Two RG patients needed complementary clinical treatment (grade II), a pancreatitis case and an ARI (acute renal injury) case. Regarding grade III, 1 RG patient presented with a late urinary fistula, treated with ureteral catheter, while 1 CG patient had a spleen injury during surgery, being managed with thermal energy and hemostatic agent only. Hospitalization staying was similar (3.3 vs. 2.7 days; p = 0.261).

Pathology results were similar between the groups, with most staged T1a, of clear cells variant, and with no positive margin at all.

Regarding participants’ functional variables, there were no statistically significant differences between groups in the changes from baseline in serum creatinine or GFR at 30, 90, and 180 days after surgery.

## DISCUSSION

In this study, 3D images were generated using Brainlab Elements and visualized via the Magic Leap 1 device. Yoshida et al. used 3D HoloLens and printed models, while Edgecube et al. applied intracorporeal AR projection (Paris system) involving a projector, receptor, and laparoscopic ultrasound (13, 14). All approaches proved feasible and reproducible.

Significant differences were observed in ischemia time and selective clamping, with the RG showing a mean ischemia time 3.8 minutes shorter than the CG (14.6 vs. 18.4 min; p = 0.045), consistent with previous findings (mean difference of 3.96 min) from a systematic review (11). While the accepted ischemia time limit is 20–25 minutes, every 1 minute saved is worthy. A retrospective study of 362 solitary kidney patients undergoing PN showed an odds ratio of 1.05 for AKI per 1-minute increase in ischemia time (12). This benefit is clearly illustrated by the regression line in a study using renal scintigraphy to assess long-term function of the operated kidney (15).

Although in PN the clamping is traditionally done in the main renal artery, it globally exposes the renal parenchyma to ischemia; selective clamping can better preserve kidney function without compromising oncologic results (16). In our study, MIXREAL use was associated with a shift toward selective clamping: 25% of RG patients underwent selective clamping versus 0% in the CG (p = 0.013). While this finding may be limited by sample size, it aligns with Piramide et al., who found a lower global ischemia rate in the 3D group despite 25.9% of the 2D group also receiving selective clamping (OR 0.22; p = 0.02) (17). Although no significant difference was found regarding off-clamp use between groups, MIXREAL may facilitate its adoption in future studies, since MIXREAL eases the understanding of the relation between the tumor and segmental vessels; the possibility to avoid ischemia at all is potentially more beneficial than selective clamping.

Percentage of residual parenchyma after surgery is as critical as ischemia time and the percentage of parenchyma subjected to ischemia for renal function recovery (18). Enucleation maximizes nephron preservation and thus renal function (19). Porpiglia et al. showed significantly higher enucleation rates when minimally invasive PN was combined with MIXREAL (9, 20), a finding supported by a meta-analysis reporting enucleation rate of 31.3% in 3D versus 18.9% in 2D groups (17). Although not statistically significant in our study, enucleation was more frequent in RG (40% vs. 20%); we can hypothesize this lack of significance to small sample and to the fact that RG tumors presented a higher trend towards high-risk RENAL score (30% vs 8%) (p = 0.124) and presence of multiple lesions (30% vs 8%) (p = 0.055).

Despite the trend toward more complex lesions in RG, complication rates were similar between RG and CG. The use of advanced tools like MIXREAL is valuable not only for managing complex tumors but also for challenging surgical scenarios, such as the dense inflammation often seen in salvage PN after ablative therapies (21). Systematic reviews also show no difference in complication rates (22), and some evidence suggests that 3D technologies may reduce the risk of collecting system entry (9). Combining MIXREAL with other strategies, such as retroperitoneal access—now more common with the spread of single-port robotic platforms (23)—may further reduce complications. For example, the spleen injury observed in our cohort might have been avoided with retroperitoneal access. Notably, a systematic review of 160 RAPN cases using single-port systems reported a low complication rate (5%) and a mean EBL of 64.25 mL (24).

In this study there was no statistical difference for TST (total surgical time). On the other hand, most evidence, with statistical significance, points out that the use of MIXREAL adds shorter surgical time, with an average of 22 minutes less (11, 25) TST and may, in fact, contribute to reduce TST.

Possibly due to the greater complexity of RM from RG, mean EBL was higher (264.6 mL vs 138.0 mL), but as well as there was no statistical significance for this outcome, there was no difference in the use rate of haemostatics, nor in the rate of transfusion, and conversion to RN only occurred in the CG (0% vs. 8%); although absence of statistical significance (p=0.495), it is important to emphasize that conversion to radical nephrectomy represents the most unfavorable scenario with respect to functional outcome, and any harmless resource available should be used to potentially avoid RN. Also, we have to consider that a mean EBL difference of 126 mL is not clinically significant. Furthermore, lower EBL rate in the context of 3D use, with statistical significance, is evidenced since the first meta-analysis that compared PN with and without the use of MIXREAL (25), which is still reproduced in more recent studies (20).

Renal injury is determined by some variables, such as resection technique, ischemia time and EBL, being quantified through the GFR. In the present study, even with RG having shorter average ischemia time and greater enucleation percentage, there was no statistical difference between groups regarding renal function variability over time. In systematic review by Jiaqi, renal function was also evaluated in 3 and 6 months, and there was no statistical difference (25). One hypothesis is that there may be a difference in the postoperative renal function in favor of MIXREAL, but that this difference is masked by compensatory effect of a healthy contralateral kidney. Some studies support this hypothesis, such as Li et al, which compared the use or not of AR in RAPN, but in single kidney patients, with those of the intervention group presenting a lower loss of renal function, with statistical significance (10). In the same line, Porpiglia et al. used DMSA scintigraphy to estimate the absolute renal function of each kidney and found a better outcome in the group submitted to AR, also with statistical significance (9).

Regarding oncologic outcomes, the use of MIXREAL had already proven to be safe, as seen in systematic reviews (26, 27); for instance, our study had no cases of positive margin. More than safe, MIXREAL possibly offers better oncologic outcomes, which can be hypothesized by the fact that there is already study showing that enucleation reduces the risk of positive surgical margin compared to nucleo-resection (28), and that the use of MIXREAL favors the chances of being able to make enucleation (9).

The high cost for the absorption of 3D systems compared to 2D systems is still one of the main reasons for the lack of broader diffusion of MIXREAL (29). In our institution, the cost for acquisition of the software, previously from the study, and of the goggle was 80,000 and 9,000 USD, respectively, while the cost of each rendering by an engineer is estimated to be 500 USD; in our study, the images were rendered by an engineer (AK) for free and by the first author, whose average time for 3D rendering after the learning curve was approximately 120 minutes.

Another disadvantage of MIXREAL technology available at the moment is that extreme renal rotations and posterior tumors still represent limitations to the superimposing of virtual images on the surgical field. It is expected that in the future, the application of artificial intelligence with "Deep Learning" algorithms may be a reliable option for renal visualization throughout the procedure (22). Nonetheless, 35% of RG lesions were located posteriorly and there was no heterogeneity with the CG in relation to the location of the tumor.

Finally, in relation to the limitations of the study it is noteworthy that the small sample size may be associated with the lack of statistical significance in several variables. Also, it is worth mentioning that the heterogeneity of the surgeons, even though all were experienced, can also impact several perioperative variables and that this was the first study using the Brainlab Elements® software for this purpose, so the 3D reconstructions are likely to improve their quality over time, which can influence statistical data.

## CONCLUSIONS

In view of all the already proven and potential benefits for the use of MIXREAL, it is expected that its use can increase PN indications and improve the nephron sparing surgery success rate. Three-dimensional models can be accessed by the surgeon for a detailed study of the case before surgery or may be used intraoperatively as both consultation and overlay in real time.

Within the limitations of this RCT, our results primarily demonstrate the feasibility and safety of MIXREAL in the setting of RAPN, as well as its potential to support intraoperative decision-making. Importantly, this is the first RCT to evaluate MIXREAL in RAPN, and the first such experience in Latin America. Yet, further studies with larger samples and longer follow-up are required to establish the possible functional benefits MIXREAL in minimally invasive PN.

## Data Availability

Uninformed

## ABBREVIATIONS

RCT: = Randomized clinical trial
MIXREAL: = Mixed reality
VR: = Virtual reality
AR: = Augmented reality
RAPN: = Robot-assisted partial nephrectomy
RM: = Renal masses
RG: = Realitatem Group
CG: = Control Group
PN: = Partial nephrectomy 3D: = Three-dimensional
IRB: = Institutional Review Board
CT: = Computerized tomography
DICOM: = Digital Imaging and Communications in Medicine
EBL: = Estimated blood loss
SPSS: = Statistical Package for the Social Sciences
SD: = Standard deviation
IQR: = Interquartile range
CH2: = Chi-square
BMI: = Body mass index
CCI: = Charlson comorbidity index
TST: = Total surgical time
RN: = Radical nephrectomy
ARI: = Acute renal injury
GFR: = Glomerular flow rate
